# Effect of weight loss orientation on BMI in obese and overweight infertile patients

**DOI:** 10.5935/1518-0557.20220049

**Published:** 2023

**Authors:** Beatriz Bacheschi do C Benetti, Mário S Approbato, Fabiana C Approbato

**Affiliations:** 1 Nutritionist, Master’s Student in Health Sciences at the Federal University of Goiás, Brazil; 2 Director of the Human Reproduction Center - Department of Obstetrics and Gynecology, Federal University of Goiás, Brazil; 3 PhD in Health Sciences from the Federal University of Goiás, Brazil

**Keywords:** infertility, body mass index (BMI), overweight, obesity

## Abstract

**Objective:**

The aim of this study was to evaluate the response to weight loss guidance in the anthropometric parameters of obesity and overweight infertile patients assisted fertilization treatment (high and low complexity).

**Methods:**

Retrospective cohort study. This survey was conducted in a population of overweight and obese infertile patients. In the first consultation at the assisted reproduction clinic (Human Reproduction Laboratory HC / UFG), obese and overweight patients were weighed, measured and instructed to lose weight and informed that being overweight could reduce the chances of success in the treatment.

**Results:**

We analyzed 56 overweight and obese patients admitted for infertility treatment at the Human Reproduction Center HC/UFG. The mean age of overweight and obese patients was 35.78 years, SD 3.70. After the orientation, only 8.92% of patients would achieve the normality rating for BMI, overweight 39.28% (decreased 14.29%), obesity I 37.5%, obesity II 10.71% and obesity III 3.57% (all degrees of obesity increased 1.79%). The mean weight of patients before and after guidance was statistically significant (*p*<0.0046). The mean values of BMI before and after guidance were also statistically significant (*p*<0.0038).

**Conclusions:**

Weight loss guidance in this population had no effect on weight loss. On the contrary, the mean weight of patients after guidance was statistically higher than the mean in the first consultation (both weight and BMI). It is suggested that for obese and overweight infertile patients, in addition to guidance for reduction, an appointment with a nutritionist and/or endocrinologist should be immediately scheduled.

## INTRODUCTION

Obesity is a worldwide epidemic and causes serious damage to public health (Loret de Mola, 2009). According to the World Health Organization, in 2016, in the world population over 18 years old, the percentage of overweight was 39% and obesity 13%. In Brazil, the percentage of overweight in men was 55.4% and in women 53.9%, and in both sexes the percentage of obesity was 20.3% (VIGITEL, 2021).

The concept of obesity is the excessive accumulation of fat in the body, which can compromise the individual’s health, as well as triggering the onset of diseases such as type II diabetes mellitus and cardiovascular diseases. Obesity can also be the cause of the development of psychosocial disorders, such as depression, body image distortions and anxiety disorders (Barbieri & Melo, 2012).

The World Health Organization classifies obesity based on the BMI defined by the calculation of body weight, in kilograms, divided by the square of height, in square meters (BMI = kg/h^2^). Overweight people are those with a BMI equal to or above 25 kg/m^2^ and less than 30 kg/m^2^. Obesity is characterized when the BMI is equal to or above 30 kg/m^2^ (WHO, 2015).

Infertility is also classified as a public health problem (WHO, 1991). Infertility is defined as the failure to conceive, without the use of contraceptives, for a period of one year with sexual intercourse for women under 35 years old and six months for women over 35 years old (ASRM, 2020). Obesity-rated women are three times more likely to suffer from infertility than normal-weight women (Brewer & Balen, 2010). Obesity can have a negative influence on a woman’s menstrual cycle, having as one of the mechanisms, the absence of ovulation (ASRM, 2008).

A cohort study found in obese and ovulatory women an increase in the time to get pregnant, for each BMI unit above 29 kg/m^2^, qualifying them as subfertile. A 5% reduction in the probability of becoming pregnant in these obese women has also been demonstrated when compared to women with eutrophic BMI (van Der Steeg *et al*., 2008).

The aim of this study was to evaluate the response to weight loss guidance in the anthropometric parameters of obesity and overweight infertile patients assisted fertilization treatment (high and low complexity).

## MATERIAL AND METHODS

Retrospective cohort study. This survey was conducted in a population of overweight and obese infertile patients. The guidance on weight reduction was provided by resident doctors and graduate students of Human Reproduction under the instruction of the faculty. Patients were also informed that if they did not reach the maximum value of 31 kg/m^2^, the patient could lose her place for treatment (a prerequisite for undergoing treatment at the Human Reproduction Laboratory HC/UFG). Patients are instructed to lose weight at the patient’s first consultation and the guidance is regarding the main consequences of overweight and obesity in the treatment of infertility, one of which is the decrease in the chances of successful treatment. The increased risk for obesity-associated complications is presented for patients in Table 1.

**Table 1:** Increased risk for obesity-associated complications - Human Reproduction Laboratory HC/UFG, 2021.

Complications	Increased risk (Odds Ratio)
Infertility	3 times^1^
Abortion	1.67 times^3^
Thromboelism	4-5 times^2^
Hypertension (pre-eclampsia)	3.87 times^2^
Stillbirth	1.24 times^3^
Caesarean	2.4-3.7 times^3^
Congenital anomalies - Spina bifda -Neural tubal defects - Limb reduction anomalies - Cardiovascular anomalies - Cleft lif and palate	2.24 times^3^ 1.87 times^3^ 1.34 times^3^ 1.30 times^3^ 1.20 times^3^
Birthweight	2 times^2^
Gestational diabetes	4-9 times^2^

1 (Brewer & Balen, 2010);

2 (Poston *et al.,* 2016);

3 (Catalano & Shankar, 2017).

In the first consultation at the assisted reproduction clinic (Human Reproduction Laboratory HC / UFG), obese and overweight patients were weighed and measured. The interval between the first and second weighing was approximately one year to two years. The BMI was a being the main parameter for assessing weight variation.

Exclusion factors (confounding variables) were patients with multifollicular ovary (PCOS or ovarian follicle count above 12 in at least one ovary), bariatric surgery, patients with BMI >41 or ≤ 18.5 in the first orientation visit and patients who refuse to participate in the work. Inclusion factors were patients undergoing IVF, patients aged between 20 and 40 years. patients with a BMI between 18.6 and 41 and patients who agreed to participate in the work. We calculated the odds ratio, with a 95% CI. The statistical test was the test t, with a *p* value of 0.05.

## RESULTS

We analyzed 56 overweight and obese patients admitted for infertility treatment at the Human Reproduction Center HC/UFG. The mean age of overweight and obese patients was 35.78 years, SD 3.70. After the orientation, only 8.92% of patients would achieve the normality rating for BMI, overweight 39.28% (decreased 14.29%), obesity I 37.5%, obesity II 10.71% and obesity III 3.57% (all degrees of obesity increased 1.79%).

Table 2 shows the mean weight of patients before and after guidance, which is statistically significant (*p*<0.0046). The mean values of BMI before and after guidance are shown in Table 3, significant diferences were also found (*p*<0.0038). Table 4 shows pre- and post-guidance changes in all BMI clas-sifications (normal, overweight, obesity I, II and III).

**Table 2:** Distribution of patients according to weight before and after weight loss recommendation - Laboratory of Human Reproduction HC/UFG, 2021.

Weight (Pre)			Weight (Post)			
n	(Kg)	sd	n	(Kg)	sd	p
56	77.11	11.77	56	78.95	12.86	0.0046

**Table 3:** Distribution of patients according to BMI values before and after weight loss guidance – Human Reproduction Laboratory HC/UFG, 2021.

BMI (Pre)			BMI (Post)			
N	(Kg/m^2^)	sd	n	(Kg/m^2^)	sd	p
56	29.69	3.75	56	30.42	4.32	0.0038

n = number; *Statistical test: t test, *p*=0.05.

**Table 4:** Distribution of patients according to BMI variation pre and post orientation of the first consultation of 56 overweight and obese patients - Laboratory of Human Reproduction HC/UFG, 2021.

	BMI pre orientation		BMI post orientation	
BMI classification	Frequency	%	Frequency	%
Normal (18.5 a 24.9 kg/m^2^)	0	0	05	8.92
Overweight (25.0 a 29.9 kg/m^2^)	30	53.57	22	39.28
Obesity I (30.0 a 34.9 kg/m^2^)	20	35.71	21	37.5
Obesity II (35.0 a 39.9 kg/m^2^)	05	8.92	06	10.71
Obesity III (>40.0 kg/m^2^)	01	1.78	02	3.57

BMI=Body Mass Index.

## DISCUSSION

In our study, it was seen in the retrospective comparison of patients’ parameters after the orientation period that most patients gained weight. Studies that performed more effective and objective lifestyle interventions, such as changing dietary patterns and starting a physical activity protocol with patients, had more promising results in terms of weight loss in a relatively short period of six months to one year.

In this line of research, a study of quality-of-life intervention was carried out in a group six months before starting treatment for infertility, and the control group that started treatment immediately, without any type of intervention, obtained an average of weight loss after six months in the intervention group was greater than in the control group (*p*<0.001) (Mutsaerts *et al*., 2016). As in the previous study, Einarsson *et al*. (2017) performed a weight loss intervention in a group of patients before undergoing IVF treatment and compared it with the control group, obtaining higher percentages of weight loss in the intervention and IVF group than in the group that only underwent IVF (*p*<0.0001). These works claim that there is insufficient evidence that programs that perform dietary and physical activity interventions before IVF for overweight and obese women improve pregnancy and live birth rates (Norman & Mol, 2018).

Sim *et al*. (2014) performed an intervention for weight loss over a period of twelve weeks. In this study, participants received a weekly diet, guidance on physical activity, psychological and behavioral monitoring regarding weight loss and infertility. The standard treatment group had only the advice to look for a doctor to help them in the process of reducing their body weight, so the responsibility was with the patient. The default group also received the same material as the other group. Both groups had weight loss, but the intervention group showed greater weight reduction (−6.6±4.6kg) than the other group (−1.6±3.6kg), the same reductions were seen in the BMI, with statistically significant values (*p*<0.001). Other diferences seen were with respect to the number of cycles needed in treatment, better fertilization rates and increases in pregnancy rates when purchasing groups.

As in the work by Sim *et al*. (2014), the control group (in that work called standard treatment), the participants were only guided about the benefits of weight reduction for better responses in the infertility treatment, in this work a low percentage (37.5%) of patients who managed to achieve this goal. On the contrary, it was seen that the majority (50%) had an increase in weight, and the others maintained their weight (12.5%) during this period. This work demonstrates that the Human Reproduction Laboratory’s weight loss orientation was ineffective, and there is a need for a more energetic intervention. Therefore, the approach to care for overweight and obese patients treated at the fertility center should be reviewed.

Also analyzing a review article, with a compilation of ffteen articles, it was found that in general that patients who are randomly allocated to lifestyle intervention groups would achieve greater reductions in body weight when purchased from those patients who underwent interventions minimal or no intervention prior to infertility treatment (Hunter *et al*., 2021).

## CONCLUSIONS

Weight loss guidance in this population had no effect on weight loss. On the contrary, the mean weight of patients after guidance was statistically higher than the mean in the first consultation (both weight and BMI). It is suggested that for obese and overweight infertile patients, in addition to guidance for reduction, an appointment with a nutritionist and/or endocrinologist should be immediately scheduled.
